# Aminooxyacetic acid attenuates post‐infarct cardiac dysfunction by balancing macrophage polarization through modulating macrophage metabolism in mice

**DOI:** 10.1111/jcmm.14972

**Published:** 2020-01-13

**Authors:** Pei Zhao, Wenjing Zhou, Yanxia Zhang, Jingjing Li, Ye Zhao, Lihua Pan, Zhenya Shen, Weiqian Chen, Jie Hui

**Affiliations:** ^1^ Department of Cardiovascular Surgery The First Affiliated Hospital of Soochow University & Institute for Cardiovascular Science, Soochow University Suzhou China; ^2^ Department of Cardiology The First Affiliated Hospital of Soochow University Suzhou China; ^3^ Department of Cardiology The Affiliated Hospital of Yangzhou University Yangzhou China

**Keywords:** aminooxyacetic acid, macrophage polarization, metabolic reprogramming, myocardial infarction, NLRP3

## Abstract

Excessive activation of pro‐inflammatory M1 macrophages following acute myocardial infarction (MI) aggravates adverse cardiac remodelling and heart dysfunction. There are two break points in the tricarboxylic acid cycle of M1 macrophages, and aspartate‐arginosuccinate shunt compensates them. Aminooxyacetic acid (AOAA) is an inhibitor of aspartate aminotransferase in the aspartate‐arginosuccinate shunt. Previous studies showed that manipulating macrophage metabolism may control macrophage polarization and inflammatory response. In this study, we aimed to clarify the effects of AOAA on macrophage metabolism and polarization and heart function after MI. In vitro, AOAA inhibited lactic acid and glycolysis and enhanced ATP levels in classically activated M1 macrophages. Besides, AOAA restrained pro‐inflammatory M1 macrophages and promoted anti‐inflammatory M2 phenotype. In vivo, MI mice were treated with AOAA or saline for three consecutive days. Remarkably, AOAA administration effectively inhibited the proportion of M1 macrophages and boosted M2‐like phenotype, which subsequently attenuated infarct size as well as improved post‐MI cardiac function. Additionally, AOAA attenuated NLRP3‐Caspase1/IL‐1β activation and decreased the release of IL‐6 and TNF‐α pro‐inflammatory cytokines and reciprocally increased IL‐10 anti‐inflammatory cytokine level in both ischaemic myocardium and M1 macrophages. In conclusion, short‐term AOAA treatment significantly improves cardiac function in mice with MI by balancing macrophage polarization through modulating macrophage metabolism and inhibiting NLRP3‐Caspase1/IL‐1β pathway.

## 1 INTRODUCTION

In the past 20 years, owing to implementation of evidence‐based treatment, the in‐hospital, 30‐day and 1‐year all‐cause mortality of acute myocardial infarction (AMI) were approximately 43%, 42% and 36% lower, respectively, in 2013‐2014 than in 1995‐1996.^1^ Even so, AMI leads to irreversible loss of myocardial cells and poor left ventricular remodelling, resulting in reduced ejection fraction (<50%, 72% in 1997‐1998 and 52% in 2013‐2014) 1 and subsequent heart failure. More importantly, the mortality seems to have remained constant and not fallen further since around 2008.^1^, ^2^ Therefore, new effective therapeutic strategies for MI are still desirable.

The infiltration of immune cells, particularly macrophages, plays important roles in poor left ventricular remodelling and subsequent heart failure after MI.^3^, ^4^ Macrophages have peculiar plasticity that allows them to functionally polarize into classically activated M1 macrophages or alternatively activated M2 macrophages in response to different stimuli, and these different types play opposite roles. Studies showed macrophages were differentially activated during different phases after MI. The M1 subtype dominates the early inflammatory phase and secretes pro‐inflammatory cytokines, causing damage, which then transits to the M2 macrophages and expresses high level of IL‐10 in the infarct scar formation stage after MI, promoting repair.^5^, ^6^, ^7^ In AMI, the transformation of pro‐inflammatory macrophages to anti‐inflammatory macrophages is considered to be the beginning of myocardial repair after MI, and the failure of the transformation may result in sustained M1 macrophages activation and adverse cardiac remodelling.^5^ Therefore, modulating the balance of pro‐inflammatory macrophages and anti‐inflammatory macrophages is speculated as a novel treatment method.^5^, ^8^

Interestingly, M1 macrophages preferentially metabolize glucose as an energy substrate, converting glucose into lactate.^9^, ^10^ However, M2 macrophages utilize fatty acids as a source of fuel, which fuel an oxidative tricarboxylic acid cycle (TCA) and following oxidative phosphorylation (OXPHOS).^10^ Recent studies have shown that enhanced glucose metabolism drives a hyper‐inflammatory response in macrophages^11^ and inhibiting glycolysis reverses pro‐inflammatory cytokines elevations,^12^ indicating that manipulating macrophage metabolism may control macrophage polarization and inflammatory response. In short, glucose metabolism is central to the function of M1 macrophages and strategies for modulating its glucose metabolism would be innovative approaches to attenuate inflammatory responses and thus address MI.

Outstandingly, in M1 macrophages, the TCA cycle metabolic flux is discontinued at isocitrate dehydrogenase (IDH) and succinate dehydrogenase, and aspartate‐arginosuccinate shunt (AASS) compensates for the breaks.^13^ Aminooxyacetic acid (AOAA) is a broad‐spectrum inhibitor of pyridoxal phosphate‐dependent enzymes, including aspartate aminotransferase (AST), which is a key enzyme of AASS. Previous studies have indicated that manipulating macrophage metabolism may control macrophage polarization and inflammatory response,^11^, ^12^ inspiring us to study AOAA potential role in macrophages. In the present study, we set out to determine the effects of AOAA on macrophage metabolism and polarization and cardiac function after MI in a mouse model.

## 2 MATERIALS AND METHODS

### 2.1 Laboratory animals

Male wild‐type C57BL/6J mice aged 8‐10 weeks were purchased from the Experimental Animal Center of the Chinese Academy of Medicine Sciences of Soochow University. All animal procedures were in conformity with the local ethics legislation of animal experimentation in the Institute for Cardiovascular Science, Soochow University, Suzhou, China. Mice were allowed free access to food and water and housed in a room with controlled temperature (22 ± 1℃) and a 12‐hour light‐dark cycle.

### 2.2 Generation of primary bone marrow‐derived macrophages (BMDMs)

Bone marrow cells were flushed out from femurs and tibias of 8‐10 weeks old male C57BL/6 mice. The bone marrow cells were resuspended, cultured and differentiated into macrophages in RPMI‐1640 supplemented with 10% FBS, 1% P/S and 20 ng/mL recombinant murine macrophage colony‐stimulating factor (PeproTech) in a humidified incubator containing 5% CO_2_ at 37℃ for 7 days. On day 7, bone marrow‐derived macrophages were harvested using Accutase (Sigma) and replated for further experimentation. BMDMs were characterized by flow cytometry analysis with antibodies specific for F4/80 and CD11b.

### 2.3 Macrophages experimental protocol

Bone marrow‐derived macrophages were treated without or with indicated concentration of AOAA (Sigma) for 1 hour, followed by 10 ng/mL LPS and 10 ng/mL IFN‐γ or 20 ng/mL recombinant murine IL‐4 (PeproTech) stimulation for 24 hours. Supernatants were harvested for pH and lactic acid measurements, and the cells were collected for ATP detection, RNA or Western blot analysis.

### 2.4 Fluorescence‐activated cell sorting (FACS) analysis

Single‐cell suspensions were incubated with anti‐F4/80‐FITC, anti‐CD11b‐APC (BioLegend) or anti‐CD86‐FITC (eBioscience) for 30 minutes on ice. After two further washes, the cells were resuspended in wash buffer (1 × PBS with 2% FBS) and assessed with a Guava Easycyte 8 flow cytometer (Millipore) as previously described.^14^ In each experiment, isotype‐matched IgG controls were used. The cells expressing CD11b and F4/80 surface markers were defined as macrophages. The CD86^+^ macrophages were categorized as classically activated M1 macrophages.

### 2.5 Measurement of lactic acid

The lactic acid level was determined using a lactic acid assay kit (Beyotime) following the instructions of the manufacturer. Briefly, the supernatant of BMDMs with various treatments was collected. After incubating with enzyme reagent and developer for 10 minutes at 37°C, the reaction was stopped and lactic acid level of the supernatant was determined by measuring absorbance at 530 nm on a multifunctional microplate reader (BIO‐TEK).

### 2.6 Measurement of extracellular acidification rate (ECAR)

To evaluate glycolysis function, we measured ECAR in BMDMs using a glycolysis assay kit (Abcam) following the manufacturer's instructions. Briefly, BMDMs were seeded in 96‐well plate at a density of 3 × 10^5^ cells/well in 200 μL culture media for 12 hours to allow cells to adhere in a CO_2_ incubator at 37°C The BMDMs were then unstimulated (M0) or stimulated with LPS/INF‐γ for 24 hours with or without 1 hour pre‐treatment with AOAA (1, 5 mmol/L). Culture media were discarded from all assay wells, and BMDMs were washed with 100 μL respiration buffer two times. About 150 μL respiration buffer was added to all wells containing cells and blank controls wells. BMDMs were incubated in a CO_2_‐free incubator at 37°C for 3 hours to purge CO_2_. About 10 μL reconstituted glycolysis assay reagent was added to each sample well, and 10 μL respiration buffer was added to blank control wells. The 96‐well plate was inserted into a multifunctional microplate reader (BIO‐TEK) pre‐set to 37°C. Glycolysis assay signal was measured at 1.5 minutes intervals for 180 minutes using excitation and emission wavelengths of 380 and 615 nm, respectively.

### 2.7 Intracellular ATP assays

Intracellular ATP content was determined by using an enhanced ATP assay kit (Beyotime) as previously described^15^ with some modifications. Briefly, cells were lysed and boiled for 2 minutes to fully release ATP, and then centrifuged at 12 000 *g* at 4°C for 5 minutes. The supernatant was added to the test plate pre‐treated with ATP detection reagent. Luminescence was measured by using a microplate reader (BIO‐TEK). However, cells lysate for protein content measurement was not boiled and the protein content was measured with the BCA protein assay kit (Takara). Finally, ATP concentration was expressed as μmol/g protein.

### 2.8 Animal experimental protocol

Myocardial infarction model was established in male C57BL/6 mice as previously described.^16^ Briefly, male mice with 8‐10 weeks old were anaesthetized by intraperitoneal injection with a mixture of 70 mg/kg ketamine and 6 mg/kg xylazine and were mechanically ventilated using a rodent ventilator attached to an endotracheal tube during the surgical procedure. Thoracotomy was performed between the 4th and 5th intercostal space to expose the heart. MI was achieved through permanent ligation of the left anterior descending coronary artery with a 6‐0 polyester suture. Finally, the thoracic wall was carefully closed. The surgeon was blinded for the mouse grouping. MI mice were allocated to intraperitoneal injection with a daily dose of 10 mg/kg BW of AOAA diluted in saline (1 mg AOAA in 1 mL saline, ie 10 mL/kg solution) or 10 mL/kg saline after operation for three consecutive days. The dose of AOAA was determined according to previously published studies.^17^, ^18^, ^19^ Mice were killed at day 3 or day 28.

### 2.9 Echocardiography

For echocardiographic acquisition, mice were anaesthetized by 1%‐1.5% isoflurane inhalation and heart rate was maintained between 350 and 450 b.p.m. Cardiac function was evaluated by transthoracic echocardiography using Vevo 2100 system (VisualSonics) with an 80 MHz probe. Hearts were imaged in two‐dimensional long‐axis view at the maximum left ventricle diameter level in mice, which is used to locate the M‐mode cursor for following M‐mode images. Left ventricle end‐diastolic diameters (LVDd) and left ventricle end‐systolic diameters (LVDs) were measured from M‐mode images taken from the parasternal short‐axis view. Left ventricle ejection fraction (EF) and fractional shortening (FS) were automatically calculated by the echocardiography software. All measurements were averaged over three consecutive cardiac cycles.

### 2.10 Histological preparation

At day 3 or day 28 post MI surgery, the animals were anaesthetized and the hearts were exposed. For pathological examination, the hearts were arrested via the left ventricle injection of 1 mL 1 mol/L KCl followed by 5 mL PBS and 10 mL 4% paraformaldehyde perfusion. Then, the hearts were carefully dissected and fixed in 4% paraformaldehyde overnight. For biochemical and molecular analysis, the hearts were perfused with PBS, and then snap‐frozen in liquid nitrogen and stored at −80°C.

### 2.11 Haematoxylin and eosin (H&E) staining

After fixed in 4% paraformaldehyde overnight, the hearts of day 3 post MI surgery were processed as standard paraffin embedded. The heart tissues were then serially sectioned at 5 μm in the left ventricle transverse direction. H&E was performed to evaluate the inflammatory cell infiltration.

### 2.12 Masson's Trichrome staining

The heart tissues of day 28 after MI were sectioned in the left ventricle transverse direction from the ligation level down to the apex. About 10 serial sections (5 μm per section) were collected every 500 μm thickness intervals. Masson's trichrome staining was performed to quantified fibrosis area in the left ventricle after MI. The images were captured using a stereoscopic microscope equipped with a trinocular optical head (SMZ745T; Nikon). Left ventricle fibrosis area and total left ventricle area of each image were measured with Image J software, and fibrosis area was expressed as a percentage of the total left ventricle area.

### 2.13 Immunofluorescence staining

For immunofluorescence, after fixed in 4% paraformaldehyde overnight, the hearts of 3 days after MI surgery were immersed in 30% sucrose for several hours, embedded in OCT compound and snap‐frozen in liquid nitrogen, and stored at −80°C. Sections (5 μm) were incubated with 3% BSA in PBST with 0.1% Triton for 1 hour at room temperature. Then, sections were incubated with primary antibodies against iNOS (1:200; Abcam), Arg1 (1:50; ProteinTech Group), TNF‐α (1:200; Abcam), CD206 (1:100; ProteinTech Group) or F4/80 (1:200; Abcam) overnight at 4°C. After washing with PBST three times for 5 minutes each, the sections were incubated with appropriate secondary antibodies (Alexa Fluor 488 AffiniPure donkey anti‐rabbit or Alexa Fluor 594 AffiniPure donkey anti‐rat [1:400; Jackson ImmunoResearch]) for 2 hours at room temperature. After triple additional washing with PBST, the sections were sealed with DAPI Fluoromount‐GTM (Yeasen) to identify nuclei and prevent fluorescence quenching. Fluorescence microscopy images were caught with a laser scanning confocal microscope (LSM880; Zeiss). We used the number of iNOS and F4/80 double‐positive cells per F4/80 positive cells at the infarcted border zone to document the M1/M polarization and used the number of Arg1 and F4/80 double‐positive cells per F4/80 positive cells to document the M2/M polarization.

### 2.14 RNA extraction and quantitative real‐time PCR (qPCR) analysis

Total RNA was isolated using TRIzol reagent and quantified with ND2000 spectrophotometer (NanoDrop Technologies). DNase I‐treated RNA was reverse transcribed into cDNA using the PrimeScript RT reagent kit (Takara), and quantitative real‐time PCR (qPCR) was carried out on a StepOne Plus real‐time PCR System (Applied Biosystems) using the SYBR Premix Ex Taq reaction mix (Takara) as previously described.^20^ The fold changes of each target gene expression relative to 18S under experimental and control conditions were calculated according to the threshold cycle (CT) as *r* = 2^−ΔΔCt^, where ΔCt = CT (target) − CT (18S) and ΔΔCt = ΔCt (experimental) − ΔCt (control). The sequences of primers are listed in Table 1.

**Table 1:** Primers used for qPCR

Gene	Species	Forward primer	Reverse primer
HK2	Mouse	TGATCGCCTGCTTATTCACGG	AACCGCCTAGAAATCTCCAGA
PFKL	Mouse	GGAGGCGAGAACATCAAGCC	CGGCCTTCCCTCGTAGTGA
PKM2	Mouse	GCCGCCTGGACATTGACTC	CCATGAGAGAAATTCAGCCGAG
6PGDH	Mouse	AGCTACATAGGAATTACGGGCAA	CCGCCATAATTGAGGGTCCAG
G6PDH	Mouse	CACAGTGGACGACATCCGAAA	AGCTACATAGGAATTACGGGCAA
GFAT1	Mouse	CAGTCGGCAGTTCTATATCAAG	CGGCAGTCGCTTCAGTCC
iNOS	Mouse	GAAGAAAACCCCTTGTGCTG	TCCAGGGATTCTGGAACATT
IL‐6	Mouse	CGTGGACCTTCCAGGATGAG	CATCTCGGAGCCTGTAGTGC
TNF‐α	Mouse	AAACCACCAAGTGGAGGAGC	ACAAGGTACAACCCATCGGC
Arg1	Mouse	CAGAAGAATGGAAGAGTCAG	CAGATATGCAGGGAGTCACC
CD206	Mouse	CTCAACCCAAGGGCTCTTCTAA	AGGTGGCCT CTTGAGGTATGTG
Ym1	Mouse	AAGAACACTGAGCTAAAAACTCTCCT	GAGACCATGGCACTGAACG
IL‐10	Mouse	GCTCTTACTGACTGGCATGAG	CGCAGCTCTAGGAGCATGTG
IL‐1β	Mouse	TGTAATGAAAGACGGCACAC	CTCCACTTTGCTCTTGACTTC
18S	Mouse	GTAACCCGTTGAACCCCATT	CCATCCAATCGGTAGTAGCG

Note

6PGDH, 6‐phosphogluconate dehydrogenase; G6PDH, glucose 6‐phosphate dehydrogenase; GFAT1, glutamine: fructose‐6‐phosphate aminotransferase 1; HK2, hexokinase 2; PFKL, phosphofructokinase live; PKM2, pyruvate kinase M2.

### 2.15 Western blotting analysis

Proteins were extracted with ice‐cold RIPA buffer supplemented with protease inhibitor cocktail tablets (Roche Diagnostics GmbH) and 1 mmol/L phenylmethylsulphonyl fluoride. Then, lysates were incubated on ice for 30 minutes and centrifuged at 9600 *g* at 4°C for 15 minutes. Equal amounts of protein (30‐50 μg protein/lane) were separated by SDS‐PAGE gels and transferred onto PVDF membranes as previously described.^21^ The membranes were blocked with 5% non‐fat milk in TBST for 1 hour at room temperature and then incubated with primary antibodies against iNOS (1:1000; Abcam), Arg1 (1:1000; ProteinTech Group), NLRP3 (1:1000; Abcam) or Caspase1 (1:1000; Abcam) overnight at 4°C on a rocking platform. The β‐tubulin (1:5000; Santa Cruz) was used as an internal control. After washing with TBST three times for 10 minutes each, the membranes were further incubated with appropriate secondary antibodies (HRP‐linked goat anti‐rabbit IgG or HRP‐linked goat anti‐mouse IgG [1:4000; Santa Cruz]) for 2 hours at room temperature. After washing three more times, the immunoblots were developed by routine enzymatic chemiluminescence. The signalling was quantified by Image J software and presented as normalized to β‐tubulin.

### 2.16 Statistical analysis

Statistical analysis was performed using the GraphPad Prism 7 software (GraphPad Software). All data were presented as mean ± SEM. Normal distribution was tested using the Shapiro‐Wilk test. Differences between two groups were compared by unpaired *t* test. More than two groups were compared by one‐way ANOVA, followed with a post hoc Tukey's multiple comparisons test. All *P* values were two‐tailed, and a *P* value <.05 was considered statistically significant.

## 3 RESULTS

### 3.1 AOAA inhibits glycolysis and enhances ATP levels in classically activated M1 macrophages

**Figure 1:**
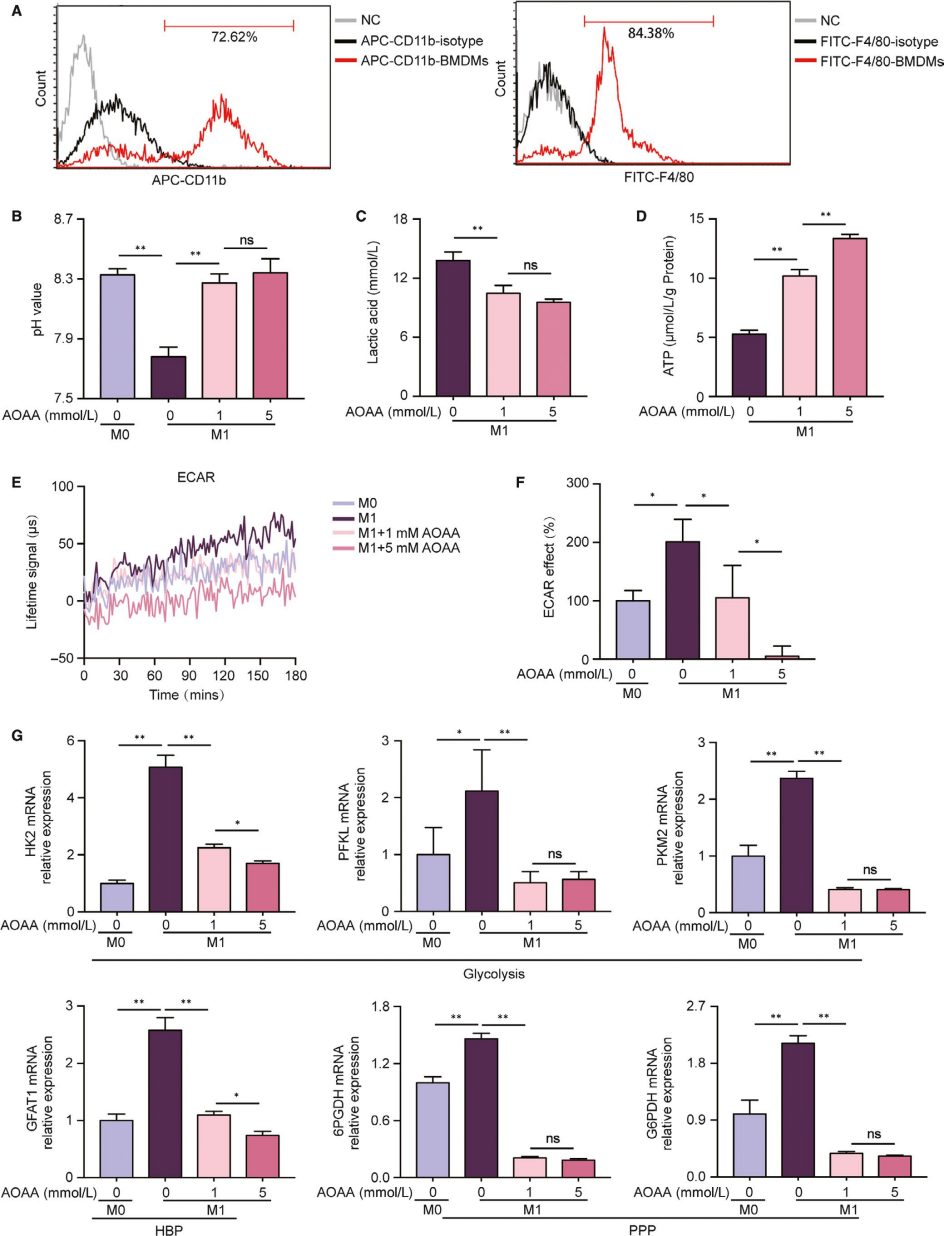
Aminooxyacetic acid (AOAA) inhibits glycolysis and enhances ATP levels in classically activated M1 macrophages. (A) FACS analysis of the macrophage surface marker CD11b and F4/80 of BMDMs. (B) pH values of BMDMs culture media, unstimulated (M0) or stimulated with LPS/INF‐γ ± AOAA (1, 5 mmol/L). (C) Lactic acid production of BMDMs after stimulation with LPS/INF‐γ ± AOAA (1, 5 mmol/L). (D) ATP level of BMDMs measured using ATP assay kit after stimulation with LPS/INF‐γ ± AOAA (1, 5 mmol/L). (E) Measurement of ECAR of BMDMs unstimulated (M0) or stimulated with LPS/INF‐γ ± AOAA (1, 5 mmol/L). (F) ECAR of BMDMs unstimulated (M0) or stimulated with LPS/INF‐γ ± AOAA (1, 5 mmol/L). (G) Gene expression profiles of limiting enzymes in glucose catabolism (HK2, PFKL, PKM2, GFAT1, 6PGDH and G6PDH) in BMDMs after stimulation with LPS/INF‐γ ± AOAA (1, 5 mmol/L). ns: no significant difference, *P < .05, **P < .01. Data are represented as mean ± SEM. HBP, hexosamine biosynthesis pathway; PPP, pentose phosphate pathway

Bone marrow‐derived macrophages were identified as the cells expressing CD11b and F4/80 surface marker by fluorescence‐activated cell sorting (FACS) analysis. As shown in Figure 1A, we found a high proportion of CD11b^+^ (71.05 ± 0.87%) and F4/80^+^ (83.04 ± 0.75%) cells, indicating that bone marrow‐derived cells isolated from the tibia and femoral of mice in our experiment were successfully differentiated into BMDMs.

The pH analysis of the macrophages culture media indicated that LPS/INF‐γ decreased the pH value and this response was reversed by 1‐hour pre‐treatment with different concentrations of AOAA (Figure 1B). Lactic acid is an important decisive factor of the pH value of cultured media. Then, we determined the effects of AOAA on the lactic acid in BMDMs under the LPS/INF‐γ stimulation. As represented in Figure 1C, 1mmol/L AOAA pre‐treatment reduced approximately 24% of lactic acid production in classically activated M1 macrophages (*P* < .01), suggesting amelioration of glucose oxidation by an enhanced flux of pyruvate into the TCA cycle. About 5 mmol/L AOAA declined the lactic acid production by 30% or more (*P* < .01), although there was no significant difference as compared with 1 mmol/L AOAA. As lactic acid is the main product of glycolysis metabolism, we further pursued the glycolysis metabolic changes of BMDMs after activating with LPS/INF‐γ for 24 hours and the effects of AOAA on the glycolysis metabolism under the LPS/INF‐γ stimulation. As illustrated in Figure 1E,F, compared with M0 macrophages, LPS/INF‐γ stimulation provoked double of ECAR, an indicator of aerobic glycolysis, indicating metabolic reprogramming towards glycolysis in BMDMs. Pre‐treatment with AOAA gradually diminished the glycolysis in a dose‐dependent manner. It is well known that a modest amount of ATP is produced in glycolysis and much more ATP is formed through TCA. Increased glycolysis in classically activated M1 macrophages means reduced ATP production. On the contrary, decreased glycolysis means raised ATP production. Therefore, we confirmed the effects of AOAA on the ATP levels in classically activated M1 macrophages. As expected, cellular ATP levels rose sharply with AOAA pre‐treatment in a dose‐dependent manner while glycolysis was inhibited (Figure 1D).

Metabolic reactions are catalysed by enzymes. What is more, not all reactions are reversible. The enzymes that catalyse irreversible reactions are rate‐limiting enzymes, which control the speed and direction of the metabolic reaction. Then, we verified expression levels of critical rate‐limiting enzymes in glucose catabolism by qPCR analysis. As illustrated in Figure 1G, there was significant increase in the expressions of HK2, PFKL, PKM2 (glycolysis), 6PGDH, G6PDH (the pentose phosphate pathway, PPP) and GFAT1 (the hexosamine biosynthesis pathway) under LPS/INF‐γ stimulation, whereas, AOAA pre‐treatment lowered the expressions of the above rate‐limiting enzymes.

Macrophages are typically divided into classically activated M1 subtype and alternatively activated M2 subtype. Classically activated M1 macrophages rely on glycolysis, whereas alternatively activated M2 macrophages acquire energy from OXPHOS.^22^ We found that AOAA seems to switch the metabolism of M1 macrophages towards M2‐like metabolism, as displayed by reduction in lactic acid production and glycolytic rates in conjunction with increment in ATP production.

### 3.2 AOAA modulates classically activated M1 macrophages phenotype and alternative activated M2 phenotype

After knowing the effects of AOAA on macrophage metabolism, we tested the effects of AOAA on macrophage polarization in the LPS/INF‐γ‐activated BMDMs.

**Figure 2:**
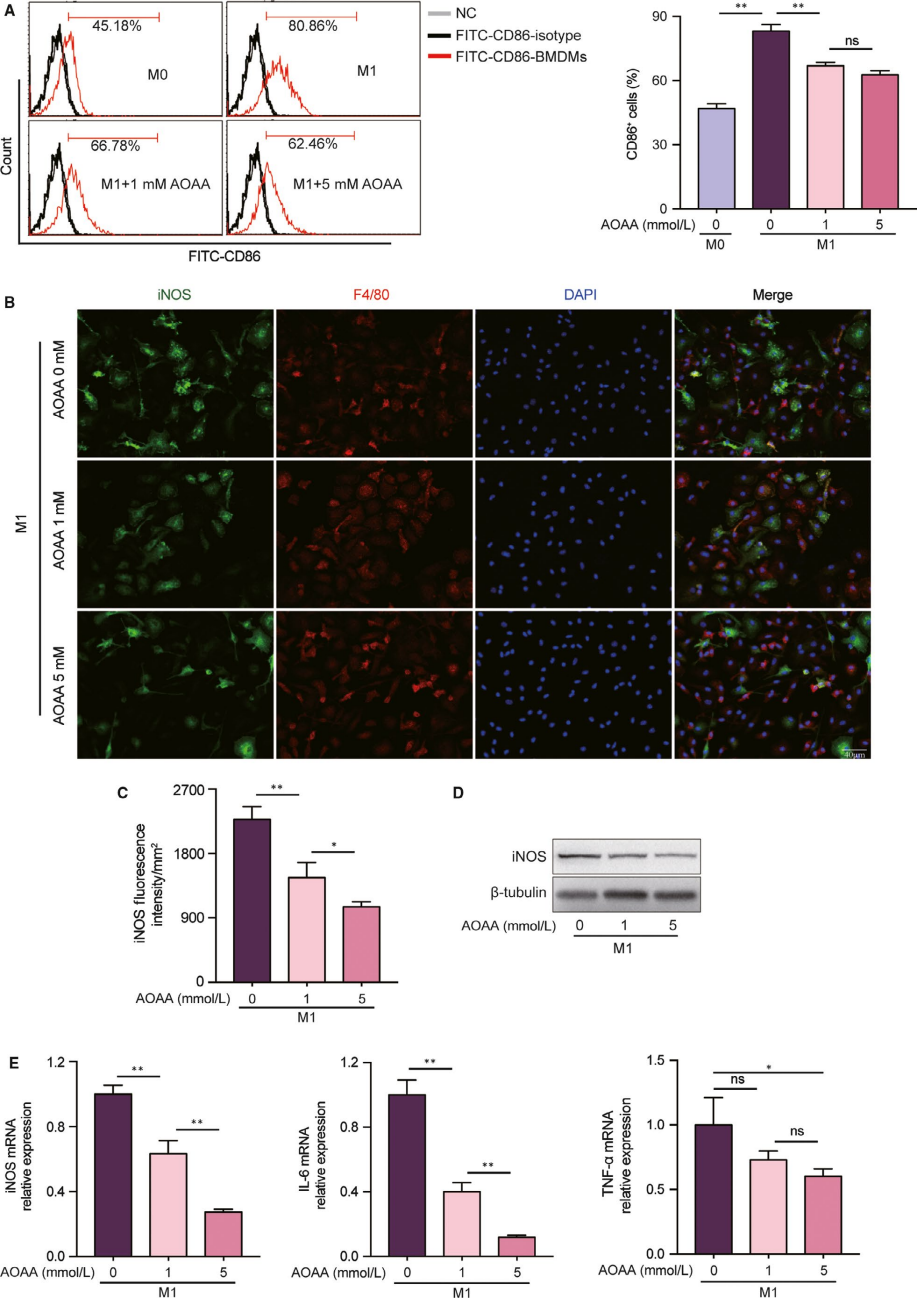
Aminooxyacetic acid (AOAA) blocks LPS/INF‐γ induced classically activated M1 macrophages phenotype and pro‐inflammatory cytokines expression in vitro. (A) Representative FACS analysis of the M1 macrophages surface marker CD86 of BMDMs and the percentages of CD86 positive cells, unstimulated (M0) or stimulated with LPS/INF‐γ ± AOAA (1, 5 mmol/L). (B) Representative confocal fluorescence imaging of iNOS in BMDMs after incubating with LPS/INF‐γ ± AOAA (1, 5 mmol/L). Scale bar equals 40 μm. (C) Quantification of iNOS Fluorescence intensity. (D) Representative immunoblot band of iNOS in BMDMs after stimulation with LPS/INF‐γ ± AOAA (1, 5 mmol/L). (E) Gene expression profiles of iNOS, IL‐6 and TNF‐α in BMDMs after stimulation with LPS/INF‐γ ± AOAA (1, 5 mmol/L). ns: no significant difference *P < .05, **P < .01. Data are represented as mean ± SEM

CD86 is a surface marker of classically activated M1 macrophages. FACS analysis indicated that after the stimulation with LPS/INF‐γ for 24 hours, CD86 positive cells reached to 83.19 ± 1.78%, which was remarkable higher than that of the macrophages without stimulation (46.87 ± 1.36%, *P* < .01). However, as indicated in Figure 2A, AOAA pre‐treatment evidently decreased the percentage of CD86 positive cells in classically activated M1 macrophages.

M1 macrophages predominantly produce pro‐inflammatory molecules such as TNF‐α, IL‐6 and iNOS. Also, iNOS is a characteristic marker of M1 phenotype. Expression of iNOS was determined by immunofluorescent staining, which showed that pre‐treatment macrophages with AOAA inhibited iNOS expression under LPS/INF‐γ stimulation in a dose‐dependent manner (Figure 2B,C). The expression of iNOS was further assessed by Western blot analysis, which revealed that the iNOS protein level was subdued when macrophages were pre‐treated with AOAA before the LPS/INF‐γ stimulation (Figure 2D).

Finally, we tested the expressions of M1 associated pro‐inflammatory cytokines by qPCR. Intriguingly, the expressions of iNOS and IL‐6 were also down‐regulated by AOAA in a concentration‐dependent manner in BMDMs under the LPS/INF‐γ stimulation (Figure 2E). However, the TNF‐α expression was frustrated only by high dose AOAA (Figure 2E).

**Figure 3:**
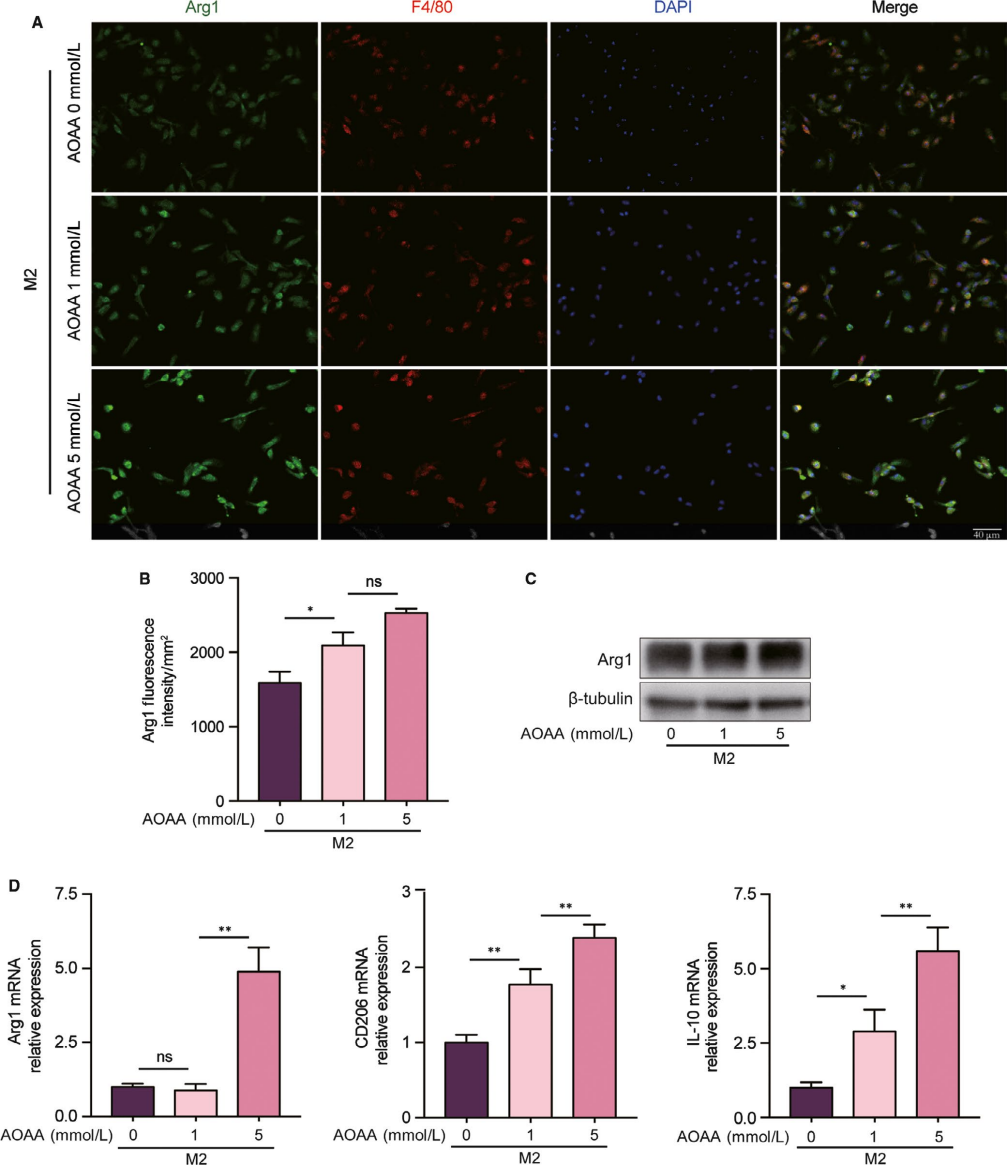
Aminooxyacetic acid (AOAA) promotes alternative active M2 macrophages polarization and anti‐inflammatory cytokine expression in vitro. (A) Representative confocal fluorescence imaging of Arg1 in BMDMs after incubation with IL‐4 ± AOAA (1, 5 mmol/L). Scale bar equals 40 μm. (B) Quantification of Arg1 Fluorescence intensity. (C) Representative immunoblot band of Arg1 in BMDMs after stimulation with IL‐4 ± AOAA (1, 5 mmol/L). (D) Gene expression profiles of Arg1, CD206 and IL‐10 in BMDMs after stimulation with IL‐4 ± AOAA (1, 5 mmol/L). ns: no significant difference, *P < .05, **P < .01. Data are represented as mean ± SEM

From the above experiments, we observed that AOAA inhibited M1 associated pro‐inflammatory cytokines expression. Since macrophages are mainly composed of classically activated M1 subtype and alternatively activated M2 subtype. We then evaluated the anti‐inflammatory properties of AOAA on M2 macrophages. Arg1 is a well‐reported and established marker of alternative activated M2 macrophages. We confirmed the effect of AOAA on the expression of Arg1 by immunofluorescent staining. We found that pre‐treatment macrophages with different concentrations of AOAA enhanced Arg1 expression under IL‐4 stimulation (Figure 3A,B). Also, the expression of Arg1 was further assessed by Western blot, which revealed that the Arg1 protein level was enhanced when macrophages were pre‐treated with AOAA before IL‐4 stimulation (Figure 3C). At last, we evaluated the expressions of M2 associated anti‐inflammatory cytokines by qPCR. We found that the expressions of CD206 and IL‐10 were dramatically increased by AOAA in a dose‐dependent manner. However, only high dose AOAA amplified the mRNA expression of Arg1 (Figure 3D).

These data suggest that AOAA adjust macrophage polarization by restraining M1 macrophages phenotype and boosting M2 phenotype, indicating a possible link between metabolic reprogramming and macrophage polarization.

### 3.3 AOAA administration attenuates post‐MI cardiac dysfunction and infarct size in mice

**Figure 4:**
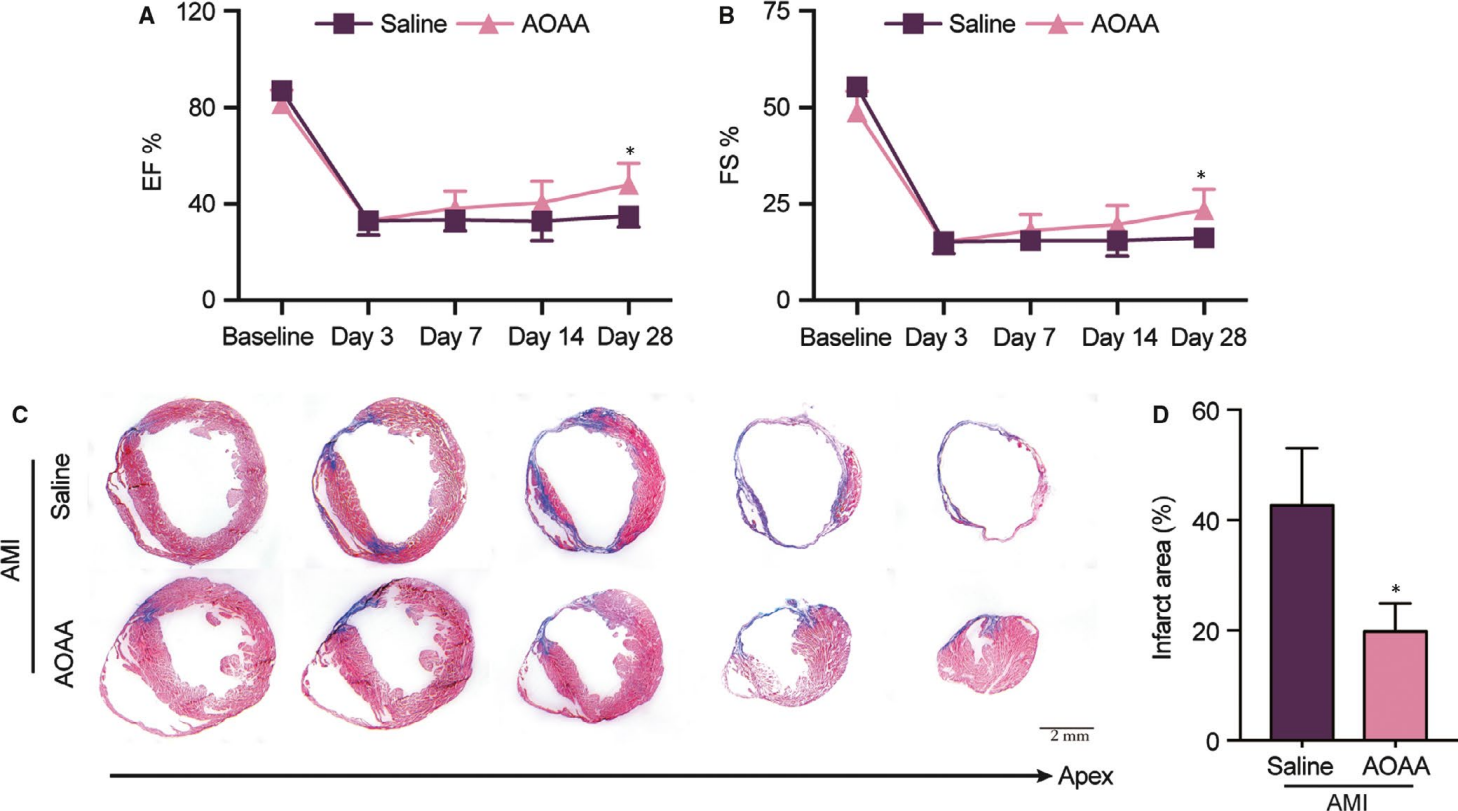
Aminooxyacetic acid (AOAA) administration attenuates post‐myocardial infarction (MI) cardiac dysfunction and infarct size in mice. (A) Ejection fraction and (B) fractional shortening of saline‐treated MI mice and AOAA‐treated MI mice were measured by echocardiography at baseline, day 3, day 7, day 14 and day 28 after MI surgery. (C) Representative cross‐sectional images of hearts stained with Masson's trichrome at day 28 post MI surgery. Serial sectioning was performed at 500 μm intervals. Scale bar equals 2 mm. Blue, scar tissue; red, viable myocardium. (D) Quantitative of the infarct percentage in heart sections following MI analysed by Masson's trichrome staining. *P < .05, Data are represented as mean ± SEM

The immune system, particularly macrophages, handle both the inflammatory and repair process after MI. Modulation of the balance between classically activated M1 macrophages and alternative activated M2 macrophages is speculated as a new treatment method.^5^, ^8^ To investigate the role of AOAA in the MI, we subjected mice to permanent ligation of the left anterior descending coronary artery. As expected, compared with baseline, EF and FS declined from 87.00 ± 0.89% and 55.36 ± 1.02% to 33.04 ± 3.02% and 15.29 ± 1.60% (all *P* < .01), respectively, at day 3 after MI, indicating that MI mice suffered from heart dysfunction. At day 28 post operation, as shown in Figure 4A,B, AOAA treatment increased 27% of EF and 32% of FS, respectively, compared with saline treatment in MI mice (all *P* < .05), suggesting an improvement of cardiac function. The results of echocardiogram were supported by histological analysis. Masson's trichrome staining revealed that AOAA reduced the infarct size relative to saline treatment (Figure 4C,D). Collectively, AOAA suppressed myocardial scar expansion and promoted cardiac function recovery from infarction.

### 3.4 AOAA reduces the proportion of M1 macrophages and boosts M2‐like phenotype in the ischaemic border zone

**Figure 5:**
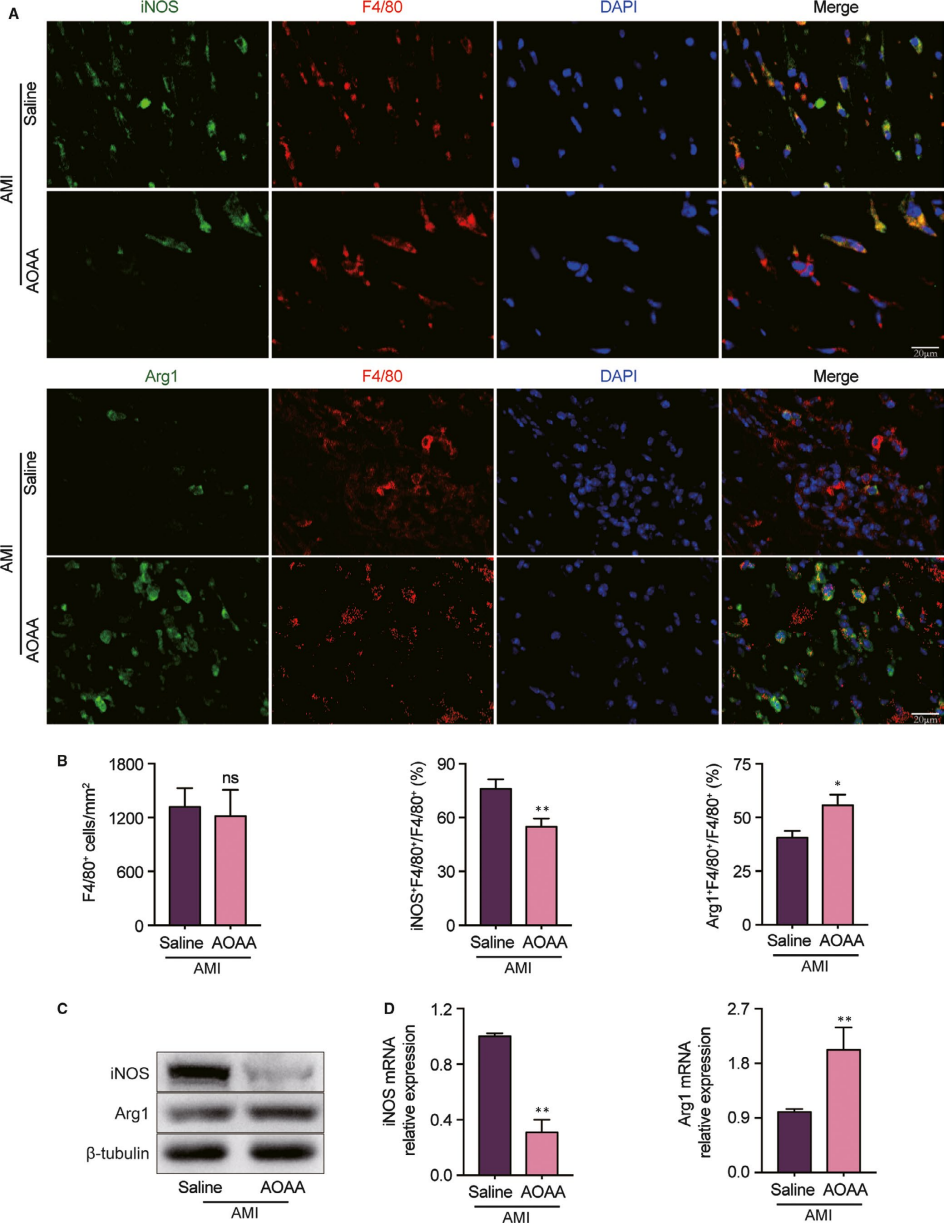
Aminooxyacetic acid (AOAA) reduces the proportion of M1 macrophages and boosts M2‐like phenotype in the ischaemic border zone. (A) Representative immunofluorescence double staining of F4/80 (red) and iNOS (green) or Arg1 (green) within the hearts of mice treated with saline or AOAA for 3 days after myocardial infarction (MI). Scale bar equals 20 μm. (B) Quantification of total macrophages (F4/80^+^), M1 macrophages (F4/80^+^iNOS^+^) and M2 macrophages (F4/80^+^Arg1^+^) within the hearts of mice treated with saline or AOAA for 3 days after MI. (C) Representative images of Western blot of iNOS and Arg1 in the hearts of saline‐treated MI mice and AOAA‐treated MI mice. (D) Gene expression profiles of iNOS and Arg1 in the hearts of mice treated with saline or AOAA for 3 days after MI. ns: no significant difference, *P < .05, **P < .01. Data are represented as mean ± SEM

To evaluate the effects of AOAA on macrophage polarization in heart tissues of MI mice, we stained heart sections with iNOS/F4/80 and Arg1/F4/80 to illustrate M1‐like and M2‐like macrophages by immunofluorescent staining. There was no significant difference in F4/80^+^ macrophages accumulation between AOAA and saline‐treated MI hearts (Figure 5A,B). However, we found that AOAA treatment reduced iNOS positive macrophages (M1‐like) proportion correlated with augmentation of Arg1 positive macrophages (M2‐like) proportion in the heart as compared with saline‐treated MI mice (Figure 5A,B). We further measured the expression of iNOS and Arg1 in the heart by Western blot and qPCR. We observed that protein and mRNA levels of iNOS were sharply attenuated by AOAA treatment compared with saline treatment in MI heart (Figure 5C,D). On the contrary, AOAA treatment led to increments of Arg1 in protein and mRNA levels in MI heart compared with those treated with saline (Figure 5C,D).

**Figure 6:**
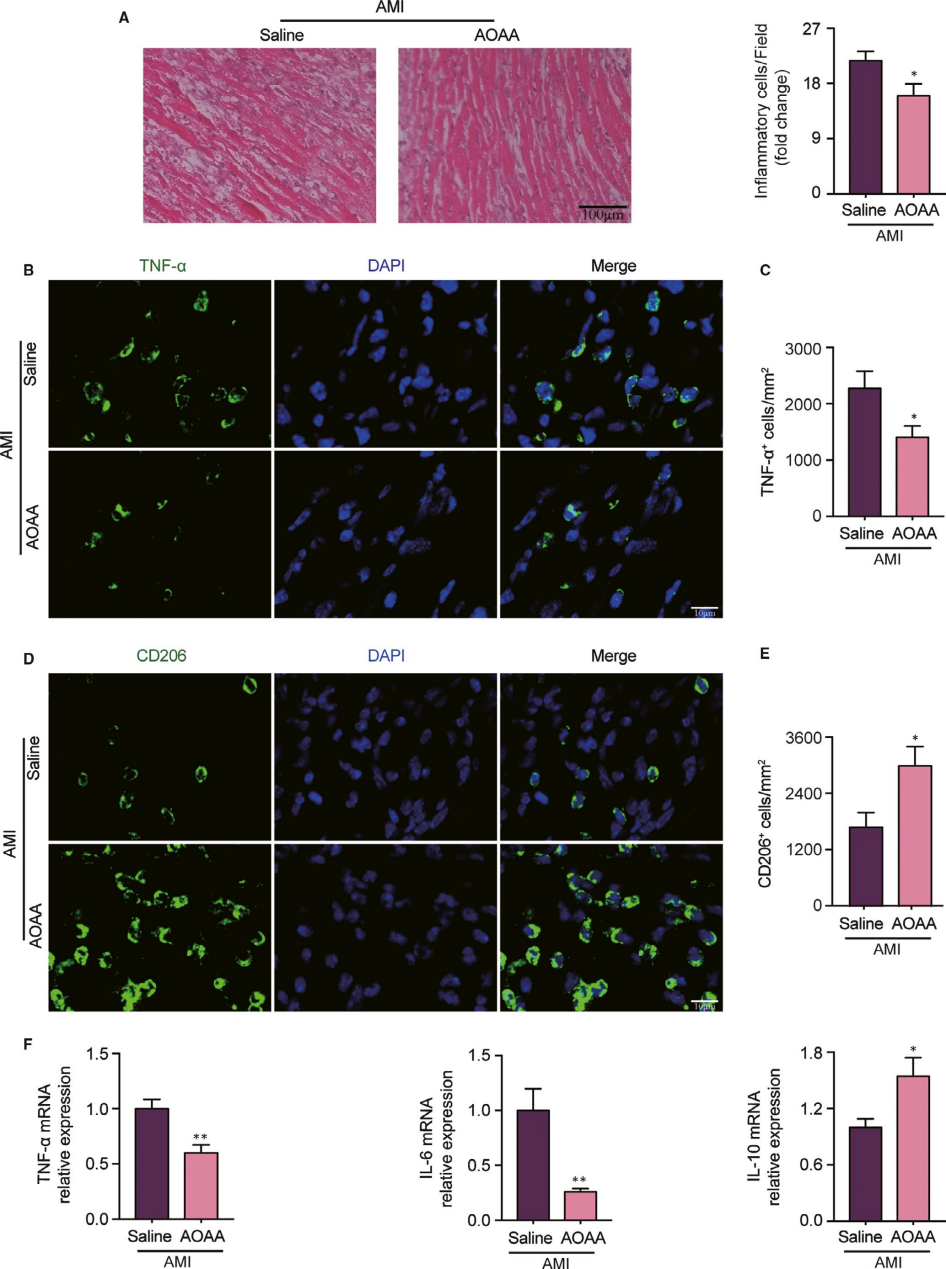
Aminooxyacetic acid (AOAA) treatment blunts cardiac inflammation response in mice with myocardial infarction (MI). (A) Phothmicrograph of representative HE staining sections and quantification of inflammatory cell infiltration (%) within the heart of mice treated with saline or AOAA for 3 days after MI. Scale bar equals 100 μm. (B) Representative confocal fluorescence imaging of TNF‐α within the hearts of mice treated with saline or AOAA for 3 days after MI. Scale bar equals 10 μm. (C) Quantification of TNF‐α positive cells. (D) Representative confocal fluorescence imaging of CD206 within the hearts of mice treated with saline or AOAA for 3 days after MI. Scale bar equals 10 μm. (E) Quantification of CD206 positive cells. (F) Gene expression profiles of TNF‐α, IL‐6 and IL‐10 in the hearts of mice treated with saline or AOAA for 3 days after MI. *P < .05, **P < .01. Data are represented as mean ± SEM

Accordingly, we assessed inflammation cell infiltration in MI heart. H&E staining analysis showed that AOAA treatment lowered the inflammatory cell infiltration in the heart tissues of day 3 after MI compared with saline treatment (Figure 6A). As indicated in Figure 6B,C, immunofluorescent staining further revealed significant TNF‐α positive signal in the peri‐infarct zones of the heart tissues, and AOAA treatment notably diminished TNF‐α positive signal. In contrast, AOAA treatment urged CD206 accumulation in MI heart manifested by immunofluorescent staining (Figure 6D,E).

To further verify the modulation of AOAA on inflammatory factor, we measured the mRNA expression of macrophage associated pro‐inflammatory and anti‐inflammatory cytokines in the heart tissues of day 3 post MI. Notably, AOAA treatment significantly inhibited the expression of TNF‐α and IL‐6 combined with a remarkable rising of IL‐10 in the heart of MI mice compared with saline treatment (Figure 6F). These data suggested that AOAA modulated macrophage polarization and early inflammation response following MI.

### 3.5 AOAA administration attenuates NLRP3‐Caspase1/IL‐1β activation in both ischaemic myocardium and LPS/INF‐γ‐stimulated BMDMs

**Figure 7:**
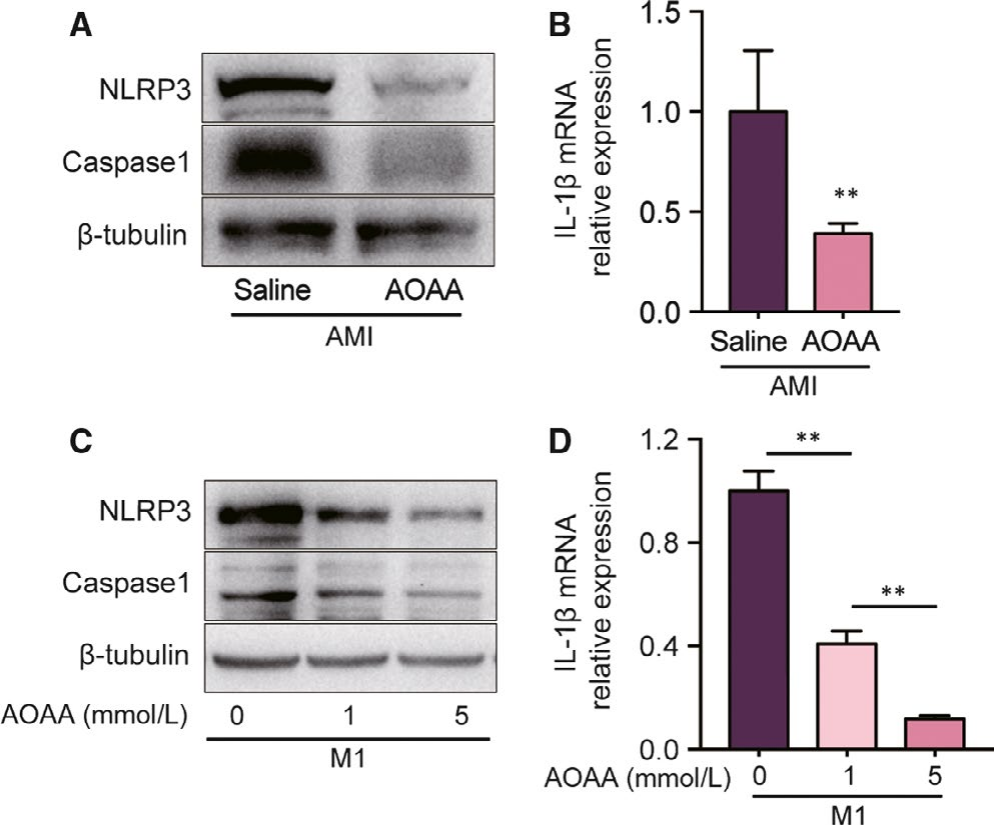
Aminooxyacetic acid (AOAA) administration attenuates NLRP3‐Caspase1/IL‐1β activation in both ischaemic myocardium and LPS/INF‐γ‐stimulated BMDMs. (A) Representative images of Western blot of NLRP3 and Caspase1 in the hearts of mice treated with saline or AOAA for 3 days after myocardial infarction (MI). (B) Gene expression profile of IL‐1β in the hearts of mice treated with saline or AOAA for 3 days after MI. (C) Representative images of Western blot of NLRP3 and Caspase1 in BMDMs after stimulation with LPS/INF‐γ ± AOAA (1, 5 mmol/L). (D) Gene expression profile of IL‐1β in BMDMs after stimulation with LPS/INF‐γ ± AOAA (1, 5 mmol/L). **P < .01. Data are represented as mean ± SEM

NLRP3 inflammasome is a multiprotein complex composed of NLRP3, ASC and Caspase‐1. It is significantly activated in MI mice,^23^, ^24^ and knockdown of its expression can reduce infarct size and improve myocardial function,^23^, ^25^ suggesting its pathophysiologic role in MI. Further, studies proved that NLRP3 inflammasome is predominantly activated in macrophages 26, 27 and up‐regulated in M1 macrophages but down‐regulated in M2.^28^ What is more, the inhibition of NLRP3 impeded glycolysis and induced a macrophage phenotype transformation from pro‐inflammatory to anti‐inflammatory.^29^, ^30^ Therefore, we investigated the effect of AOAA on NLRP3‐Caspase1/IL‐1β signalling pathway by Western blotting and qPCR analysis. As expected, AOAA treatment restrained the activation of NLRP3 and Caspase1 in the heart of the MI mice (Figure 7A). In addition, AOAA treatment dramatically constrained the IL‐1β mRNA expression in the heart of the MI mice (Figure 7B). These findings were also confirmed in vitro. We observed AOAA pre‐treatment inhibited the NLRP3 and Caspase1 protein levels in BMDMs under the LPS/INF‐γ stimulation in a dose‐dependent manner (Figure 7C). Besides, AOAA greatly frustrated IL‐1β expression in classically activated M1 macrophages in a dose‐dependent manner (Figure 7D).

## 4 DISCUSSION

In the current study, AOAA, an inhibitor of AST, modulated the balance of classically activated M1 macrophages and alternative activated M2 phenotype by regulating macrophage metabolism, and thus diminished infarct size and cardiac dysfunction. Besides, AOAA may also modulate macrophage polarization partly via attenuating NLRP3‐Caspase1/IL‐1β pathway activation in vivo and in vitro. Our findings provide novel insights to develop a feasible strategy to improve heart healing in MI patients. We confirmed and extended previous reports about the modulating role of metabolic rewiring on macrophage polarization 11, 12, 13 and the role of macrophages in AMI.^5^, ^6^, ^7^

**Figure 8:**
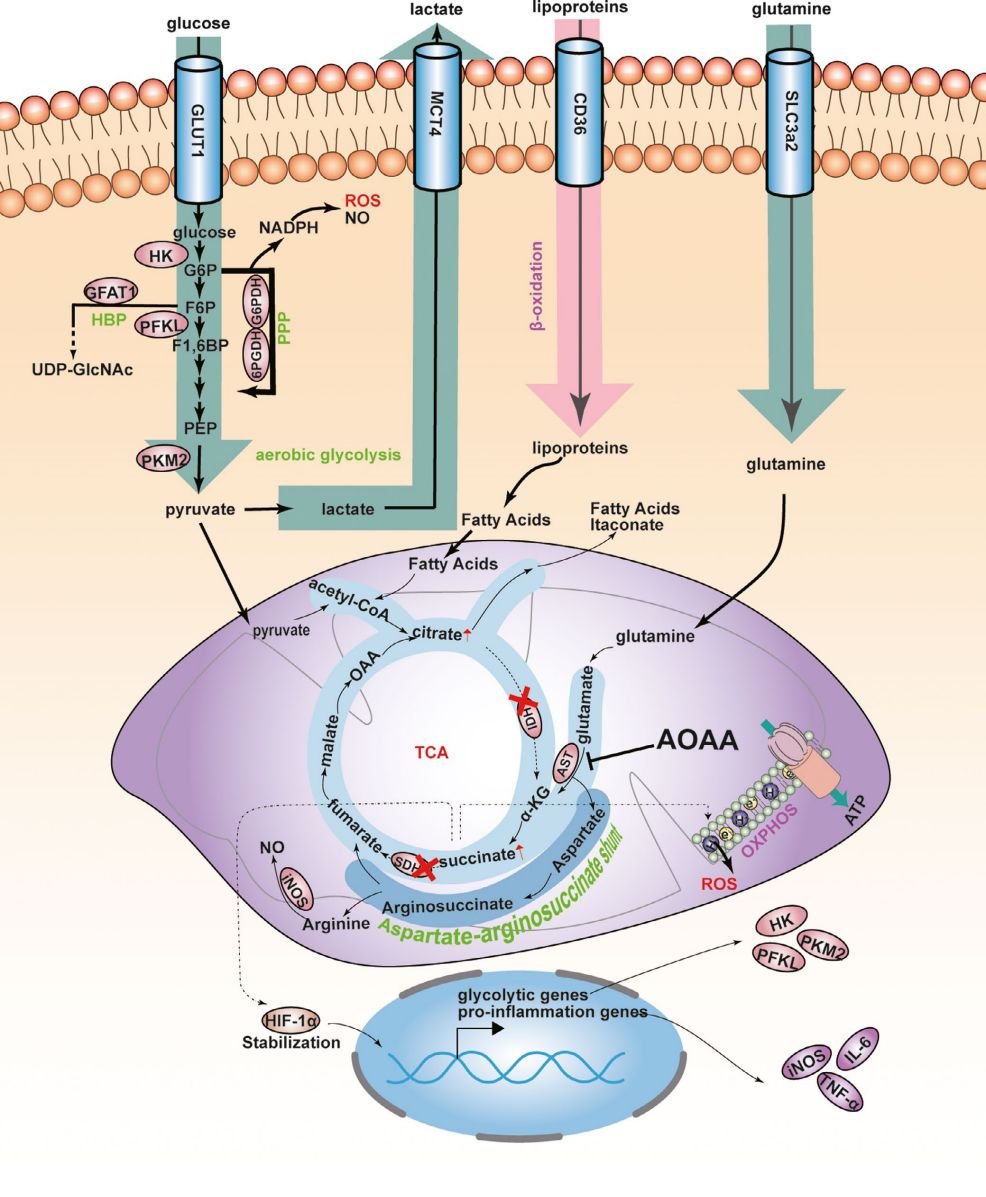
A schematic illustration on possible roles of aminooxyacetic acid (AOAA) on macrophage metabolism and polarization. Two break points lie in TCA of M1 macrophages. Aspartate‐arginosuccinate shunt compensates them. AOAA hinders succinate accumulation by inhibiting AST, resulting in the transcription inhibition of glycolysis and pro‐inflammation genes and the inhibition of ROS generating. 6PGDH, 6‐phosphogluconate dehydrogenase; AST, aspartate aminotransferase; F1,6BP, fructose‐1,6 diphosphate; F6P, fructose‐6‐phosphate; G6P, glucose‐6‐phosphate; G6PDH, glucose 6‐phosphate dehydrogenase; GFAT1, glutamine: fructose‐6‐phosphate aminotransferase 1; HBP, hexosamine biosynthetic pathway; HK, hexokinase; IDH, isocitrate dehydrogenase; NO, nitric oxide; OAA, oxaloacetate; OXPHOS, oxidative phosphorylation; PEP, phosphoenolpyruvate; PFKL, phosphofructokinase liver; PKM2, pyruvate kinase M2; PPP, pentose phosphate pathway; ROS, reactive oxygen species; SDH, succinate dehydrogenase; TCA, tricarboxylic acid cycle; α‐KG, α‐ketoglutarate

As illustrated in Figure 8, there are two break points in the TCA cycle of M1 macrophages. The broken TCA cycle leads to the failure of citrate to α‐ketoglutarate and succinate, resulting in the accumulation of citrate.^10^ In turn, AASS compensates for the breaks and becomes the major source of succinate. The mitochondria succinate can transport into the cytosol to facilitate HIF‐1α stabilization, resulting in the transcription of glycolytic and pro‐inflammatory genes.^10^ AST is a key enzyme of AASS. As an inhibitor of AST, AOAA may theoretically inhibit succinate from AASS, resulting in transcription inhibition of glycolysis and pro‐inflammation genes (Figure 8). Our data showed that AOAA decreased glycolysis rates. This is in agreement with a previous study performed in C6 glioma cells demonstrating an inhibiting role of AOAA for glycolysis rates by inhibiting AST, leading to selective disruption of glioma cells.^31^ M1 macrophages are characterized by enhanced aerobic glycolysis (converting glucose into lactate) and increased PPP flux (Figure 8). In contrast, M2 macrophages produce ATP primarily through the TCA cycle followed by OXPHOS. Besides, M2 macrophages show lower glycolysis and PPP than M1 macrophages.^13^ In this study, we found AOAA reduced lactic acid production, inhibited PPP, and increased ATP production, suggesting a switch from the metabolism of M1 macrophages to M2‐like metabolism. Further, we tested the effects of this metabolic switch on macrophage polarization in the LPS/INF‐γ‐activated BMDMs. Our results indicated that AOAA restrained M1 macrophages phenotype and reciprocally facilitated M2 phenotype. Consistently, previous evidence indicated that increasing glucose uptake up‐regulates glycolytic rates and the PPP and induces a pro‐inflammatory response.^11^ Conversely, blockade of lactate production and G6PDH (a rate‐limiting enzyme of the PPP) significantly reduced LPS‐induced TNF‐α and IL‐6 production in RAW264.7 and primary macrophages.^12^ These results confirm a pivotal role of macrophage metabolic rewiring on M1 polarization and function.

Macrophages play a key role in the pathobiology of MI. Many strategies have been proven to improve heart function after MI by modulating macrophage polarization and functions directly or indirectly.^32^, ^33^, ^34^ Based on the modulation of AOAA on macrophage polarization, we assessed the effects of AOAA in post‐MI myocardial repair. Our results indicated that the suppressing M1 polarization but boosting M2 polarization with AOAA seems beneficial for attenuating infarct size and cardiac dysfunction from MI. Collectively, these studies suggested that the modulation of macrophage polarization may be a therapeutic strategy to improve outcome after MI.

NLRP3 inflammasome has been shown to be activated in MI mice 23, 24 and knockdown of its expression can reduce infarct size and improve myocardial function.^23^, ^25^ Besides, NLRP3 inflammasome has been proved to be activated predominantly in macrophages.^26^, ^27^ Studies showed that ROS derived from NADPH oxidase and mitochondria are involved in NLRP3 inflammasome activation, and inhibition of ROS prevents the activation of NLRP3 inflammasome.^35^, ^36^ However, the exact mechanism is not clear. The structure of NLRP3 contains a highly conserved disulphide bond, which is highly sensitive to redox states.^37^ It is speculated that NLRP3 inflammasome is activated upon the disulphide bond triggered by ROS. A wide range of stimuli are also supposed to activate NLRP3 inflammasome by producing ROS.^38^ In M1 macrophages, as shown in Figure 8, ROS are mainly derived from enhanced PPP and mitochondria. And in mitochondria, accumulated succinate favours excessive ROS generation by reverse electron transport at complex I.^39^, ^40^ In the present study, the expressions of NLRP3, Caspase1 and IL‐1β were all inhibited by AOAA both in MI mice and in LPS/INF‐γ‐induced BMDMs. Therefore, we supposed that AOAA may restrain the NLRP3‐Caspase1/IL‐1β pathway by curbing ROS production though inhibiting enhanced PPP and accumulated succinate from AASS.

Xu et al^41^ noted that AOAA ameliorated experimental autoimmune encephalomyelitis in mice through shifting the differentiation of pro‐inflammatory T helper 17 cells towards anti‐inflammatory regulatory T cells. Our data confirmed that AOAA also has an anti‐inflammatory effect in MI. Meanwhile, our study showed that AOAA treatment decreased the infarct size and improved the heart function after MI by inhibiting AASS in macrophages, thus modulating macrophage polarization and inhibiting NLRP3‐Caspase/IL‐1β pathway activation. Remarkably, AASS feeds into the TCA cycle at fumarate and increases fumarate accumulation (Figure 8). Recently, fumarate has been shown to have an immunomodulatory effect. A study by McGuire et al 42 demonstrated that fumarate inhibits the transcription of pro‐inflammatory genes in M1 macrophages. Animal experiment further showed that fumarate protects the heart from ischaemia‐reperfusion injury.^43^ Therefore, the role of AASS in ischaemic myocardium is complexed and needs further elucidation in the future.

We selected AOAA in our study for its strong inhibitory effect on AST.^44^ However, it is a non‐specific inhibitor of AST, since it can inhibit several pyridoxal phosphate‐dependent enzymes.^44^, ^45^ We also could not eliminate the possibility of other mechanisms. For example, a recent study by Jespersen and colleagues showed pre‐ischaemic administration of AOAA reduced infarct size and protected the heart against ischaemia‐reperfusion injury. And, the authors owed its cardioprotection to the inhibiting of malate‐aspartate shuttle.^46^ Therefore, it is warranted to further investigate the cardioprotective mechanisms of AOAA using specific inhibitors of different enzymes separately.

In conclusion, short‐term AOAA treatment during the peak inflammatory phase of the immune response significantly improves cardiac function in mouse with MI, and its effect may be achieved by balancing the macrophage polarization, especially through modulating macrophage metabolism and inhibiting NLRP3‐Caspase1/IL‐1β pathway.

## CONFLICT OF INTEREST

The authors confirm that there are no conflicts of interest.

## AUTHOR CONTRIBUTIONS

Pei Zhao, Zhenya Shen, Weiqian Chen and Jie Hui conceived the study and designed the experiments; Pei Zhao, Wenjing Zhou and Yanxia Zhang performed the experiments and carried out the analysis; Pei Zhao drafted the manuscript; Jingjing Li and Ye Zhao constructed MI models in mice; Weiqian Chen and Lihua Pan analysed data and revised manuscript; Zhenya Shen and Weiqian Chen obtained funding and directed the project. All the authors have contributed to and approved the manuscript.

## Data Availability

The raw data supporting the conclusion of this manuscript will be made available by the authors, without undue reservation, to any qualified researcher.
